# FINANCIAL AND HEALTH INSURANCE STATUSES OF ALZHEIMER’S DISEASE AND RELATED DEMENTIA CAREGIVERS

**DOI:** 10.1093/geroni/igad104.3356

**Published:** 2023-12-21

**Authors:** Jenny Walker, Roshni Patel, Lisa McGuire

**Affiliations:** Centers for Disease Control and Prevention, Atlanta, Georgia, United States; Cyberdata Technologies (CDC Contractor), Atlanta, Georgia, United States; Centers for Disease Control and Prevention, Atlanta, Georgia, United States

## Abstract

The number of individuals with Alzheimer’s disease and related dementias (ADRD) is rising as the U.S. population ages. Important sources of care for these individuals are the approximately 11 million caregivers who donate upwards of 18 billion hours of unpaid time while incurring many of the daily expenses and out-of-pocket healthcare costs associated with their role (Alzheimer’s Association, 2023). Therefore, the financial statuses of these individuals are important to understand as we continue to develop and fortify ADRD programs and health services. Participants from 40 U.S. states and territories who completed the 2021 Behavioral Risk Factor Surveillance System caregiving module were divided into three categories: non-caregivers (n = 184,709), non-ADRD caregivers (n = 36,459), and ADRD caregivers (n = 10,583). Results from a series of Rao-Scott chi-squared tests indicate that household income levels were similar across these three categories, but a significantly higher percentage of caregivers had other people—likely the care recipient— living in their household (87.3% non-ADRD, 86.8% ADRD, 81.1% non-caregivers). Moreover, while significantly fewer caregivers reported that they lacked health coverage (7.3% non-ADRD, 6.4% ADRD) than non-caregivers (8.4%), a larger percentage reported missing a healthcare appointment in the previous year because they could not afford it (12.6% non-ADRD, 12.8% ADRD, 8.8% non-caregivers). These results further highlight the need for focused federal, state, and local interventions for community-dwelling caregiver populations to support the financial toll of caregiving.

